# Personality-Related Characteristics, Cultural Beliefs, and Labor Pain Perception After the 2023 Türkiye Earthquakes: A Prospective Study in Hatay

**DOI:** 10.3390/healthcare14131827

**Published:** 2026-06-23

**Authors:** Esra Akın, Gülay Rathfisch, Meserret Aslan

**Affiliations:** Department of Midwifery, Faculty of Health Sciences, Istanbul Atlas University, Istanbul 34408, Türkiye; akinesra1997@gmail.com (E.A.); gulay.rathfisch@atlas.edu.tr (G.R.)

**Keywords:** labor pain, childbirth, personality-related characteristics, cultural beliefs, earthquake, disaster, midwifery care

## Abstract

**Highlights:**

**What are the main findings?**

- Labor pain intensity increased progressively from 6 cm to full cervical dilatation among women giving birth in Hatay.
- In the adjusted model, education level, Extraversion, traditional-rule importance, antenatal education attendance, previous birth history and mode, and current residence were associated with labor pain scores.

**What are the implications of the main findings?**

- Labor pain should be assessed not only as a physiological experience but also through cultural, psychological, and contextual dimensions.
- Midwifery care in disaster-affected regions should be individualized, culturally sensitive, and trauma-informed.

**Abstract:**

Background/Objectives: Labor pain is a multidimensional experience associated with physiological, cultural, psychological, and contextual factors. This study aimed to examine the association of personality-related characteristics, cultural beliefs, obstetric characteristics, and proxy indicators of post-disaster context with labor pain perception among women giving birth in Hatay after the 2023 Türkiye earthquakes. Methods: This prospective observational study was conducted with 314 women admitted to Hatay Training and Research Hospital between February and June 2025. Participants were between 38 and 42 gestational weeks, had a singleton healthy fetus, were admitted in active labor, and were expected to give birth vaginally. Data were collected using a researcher-developed questionnaire, the Ten-Item Personality Inventory, and the Visual Analog Scale. Labor pain was assessed at 6 cm, 8 cm, and full cervical dilatation (10 cm). Results: VAS scores increased significantly across cervical dilatation points, from 5.04 ± 0.81 at 6 cm to 7.01 ± 0.82 at 8 cm and 8.06 ± 0.93 at full cervical dilatation (10 cm). Repeated-measures ANOVA showed a significant within-person increase in pain intensity across the three assessment points, F(2, 626) = 996.444, *p* < 0.001, partial η^2^ = 0.761. Age was not significantly correlated with VAS pain score at full cervical dilatation. In exploratory unadjusted comparisons, VAS scores at full cervical dilatation differed according to education level, official marriage status, previous birth history and mode, attendance at antenatal education, and praying to relieve labor pain. In the multivariable regression model, higher Extraversion and higher education level were associated with lower VAS scores, whereas attendance at antenatal education, greater importance given to traditional rules, previous assisted vaginal/cesarean birth, and current place of residence were independently associated with VAS scores. Conscientiousness was not significantly associated with VAS scores in the adjusted model. Earthquake experience was not significantly associated with VAS scores. Conclusions: Labor pain perception was associated with selected sociodemographic, obstetric, and cultural characteristics. The findings support the importance of individualized, culturally sensitive, and trauma-informed midwifery care in disaster-affected regions. Personality-related findings should be interpreted cautiously because the corrected reliability analysis showed low internal consistency for Agreeableness, Emotional Stability, and Openness to Experience, although Extraversion showed high internal consistency and Conscientiousness showed relatively better but still limited internal consistency. Disaster-related findings should also be interpreted cautiously because post-disaster context was assessed using only limited proxy indicators; current place of residence was independently associated with VAS scores in the adjusted model, whereas earthquake experience was not. Because of the observational design, causal interpretations cannot be made.

## 1. Introduction

Labor pain is one of the most intense and complex forms of pain experienced by women during the reproductive period [1]. Although it is rooted in physiological mechanisms such as uterine contractions, cervical dilatation, tissue stretching, and fetal descent, the way in which women perceive, interpret, and express labor pain cannot be explained solely by biological processes [2]. Labor pain is a multidimensional experience shaped by psychological characteristics, previous birth experiences, cultural beliefs, social expectations, environmental conditions, and the quality of support received during childbirth. Recent evidence emphasizes that the expression of labor-associated pain is influenced by internal factors such as fear, anxiety, obstetric history, emotional condition, expectations regarding childbirth, and the meaning attributed to pregnancy and birth. Cultural identity, social norms, language, and support during labor may also shape how pain is expressed and managed as labor progresses [3].

Pain perception is closely associated with individual psychological characteristics, particularly personality-related characteristics that shape cognitive appraisal, emotional regulation, coping behaviors, and interpersonal communication during stressful or threatening bodily experiences [4]. Within the Big Five framework, personality dimensions such as extraversion, agreeableness, conscientiousness, emotional stability/neuroticism, and openness to experience may influence how individuals interpret pain, tolerate discomfort, seek support, and express distress [5]. Neuroticism, which reflects a tendency toward anxiety, emotional reactivity, and negative affect, has been linked to greater pain-related distress and lower pain tolerance, whereas emotional stability may support more adaptive regulation of fear and discomfort. Extraversion may facilitate verbal expression of pain, communication with healthcare professionals, and the use of social support, while conscientiousness and openness may contribute to more active, organized, or flexible coping responses [6]. However, these associations should not be interpreted deterministically, because the relationship between personality and pain perception is complex and may vary according to pain type, psychological vulnerability, coping style, and clinical context [7].

In childbirth, personality-related characteristics may be particularly relevant because labor requires rapid psychological and physiological adaptation to pain, uncertainty, perceived loss of control, exposure to unfamiliar clinical procedures, and continuous interaction with healthcare professionals. Fear of childbirth is a complex construct that includes concerns about pain, injury, loss of control, abandonment, medical interventions, and the well-being of the infant, and it has been associated with women’s emotional well-being, birth preferences, and requests for cesarean birth [8]. Recent systematic review and meta-analysis evidence indicates that Big Five personality-related characteristics are significantly associated with fear of childbirth; neuroticism shows a positive association, whereas extraversion, agreeableness, conscientiousness, and openness show negative associations with fear of childbirth [9]. Fear of childbirth is also clinically relevant because it has been linked to more severe labor pain, increased analgesia use, prolonged labor, and higher likelihood of cesarean birth [10]. Therefore, examining personality-related characteristics together with labor pain perception may help describe how women exposed to similar obstetric conditions may report different levels of pain intensity.

Culture is another central determinant of labor pain perception, because cultural beliefs may shape whether pain is interpreted as a natural, meaningful, sacred, necessary, shameful, frightening, or avoidable part of childbirth. The meaning attributed to labor pain is important because women’s responses to pain may differ depending on whether pain is perceived as a sign of physiological progress, a threat, suffering, loss of control, or a meaningful transition to motherhood [11]. In some sociocultural contexts, enduring labor pain may be associated with motherhood, moral strength, religious meaning, femininity, or social value; in others, pain may be viewed primarily as suffering that should be reduced through medical or non-pharmacological support. Cultural expectations may also influence whether women feel free to express pain, ask for help, accept touch, request analgesia, or interact comfortably with healthcare professionals. A study examining cultural influence on the expression of labor-associated pain found that cultural identity, language barriers, medical care received, and companionship during birth influenced pain expression, particularly as labor progressed into more intense phases [3]. Similarly, research on cultural conceptions of labor pain and labor pain management has shown that beliefs about the value of experiencing labor pain and attitudes toward medical pain relief are socially patterned and may shape women’s expectations, preferences, and care-seeking behaviors during childbirth [12].

Personality-related characteristics and cultural beliefs may jointly shape women’s perception and expression of labor pain. Personality-related characteristics can influence cognitive appraisal, emotional regulation, coping style, and help-seeking behavior, whereas cultural norms may determine whether labor pain is perceived as a sign of strength, womanhood, suffering, vulnerability, or a condition requiring pain relief. Qualitative evidence from Ghana shows that some women are socialized to endure labor pain as a symbol of womanhood and pride, while verbal pain expression may be interpreted as emotional weakness in certain cultural contexts. This indicates that pain behavior during childbirth is not only individual but also socially learned and culturally regulated [13].

The childbirth environment may further intensify these individual and sociocultural influences. After the 2023 Türkiye earthquakes, women in affected provinces experienced displacement, temporary settlement conditions, reduced privacy, disruption of health services, and altered access to reproductive care. WHO reported that in Hatay, tents and containers became living spaces for displaced people, including pregnant women and new mothers, and emphasized the need to restore reproductive and women’s health services in the region [14]. UNFPA also reported that 226,000 pregnant women were directly affected by the earthquakes in Türkiye and that damage to health infrastructure disrupted access to sexual and reproductive health services [15].

The 2023 Türkiye earthquakes created substantial disruption in housing, privacy, social support, and access to reproductive healthcare in severely affected provinces such as Hatay. Evidence from Türkiye indicates that stress, post-traumatic stress symptoms, traumatic childbirth perception, anxiety, and depression were interrelated among pregnant and postpartum women living in post-earthquake temporary settlements [16]. These findings suggest that childbirth in disaster-affected regions should be interpreted within a broader psychosocial and contextual framework. However, in the present study, post-disaster context was assessed using only two proxy indicators: earthquake experience and current place of residence.

Despite increasing interest in psychosocial determinants of childbirth, limited evidence has examined how personality-related characteristics, cultural beliefs, obstetric characteristics, and proxy indicators of post-disaster context are associated with labor pain perception within the same observational framework. This gap is particularly important in Hatay, where cultural diversity and earthquake-related disruption coexist. Moreover, previous studies have generally focused on fear of childbirth, social support, or specific pain-management interventions, whereas fewer studies have evaluated how personality-related dimensions and culturally shaped beliefs may relate to labor pain perception during the active and transition phases of labor. Therefore, this prospective observational study aimed to examine the association of personality-related characteristics, cultural beliefs, selected obstetric characteristics, and proxy indicators of post-disaster context with labor pain perception among women giving birth in Hatay.

### Research Questions

1. What are the levels of perceived labor pain among women giving birth in Hatay during the active and transition phases of labor?
2. Are personality-related characteristics associated with labor pain perception among women giving birth in Hatay?
3. Are cultural beliefs and childbirth-related attitudes associated with labor pain perception?
4. Are the two proxy indicators of post-disaster context, earthquake experience and current place of residence, associated with personality-related characteristics and labor pain perception?
5. How are personality-related characteristics, cultural beliefs, and proxy indicators of post-disaster context descriptively related to women’s labor pain perception in a post-earthquake setting?

## 2. Materials and Methods

### 2.1. Aim and Design of the Study

This study was conducted to examine the association of personality-related characteristics, cultural beliefs, selected obstetric characteristics, and proxy indicators of post-disaster context with labor pain perception among women giving birth in Hatay. A prospective observational design was used. Labor pain intensity was assessed prospectively at three cervical dilatation points: 6 cm, 8 cm, and full cervical dilatation (10 cm). These three assessment points were selected because they represent clinically meaningful stages of labor progression and allow evaluation of pain trajectory from active labor to full cervical dilatation.

### 2.2. Population and Sample

The study population consisted of women who presented for vaginal birth at Hatay Training and Research Hospital in Antakya, Hatay. According to hospital records, the annual number of vaginal births at the hospital was 1482. The minimum sample size was calculated using the known-population sample size formula. Since the study had an observational design and no prior local estimate was available for the main outcome in this population, the known-population sample size formula was used. Based on an annual population size of 1482, a 95% confidence level, a 5% margin of error, and an assumed prevalence of 50%, the minimum required sample size was calculated as 305. The sample size was calculated using the following formula:

$$n\;=\;\frac{1482\;\cdot \;{\left(1.96\right)}^{2}\;\cdot \;0.50\;\cdot \;0.50}{{\left(0.05\right)}^{2}\;\cdot \;\left(1482-1\right)\;+\;{\left(1.96\right)}^{2}\;\cdot \;0.50\;\cdot \;0.50}\;=\;304.85$$

In this formula, N = 1482, Z = 1.96, *p* = 0.50, q = 0.50, and d = 0.05. Accordingly, the minimum sample size was calculated as 304.85, indicating that at least 305 women were required. The study was completed with 314 women. Because no prior local estimate was available for labor pain perception in this specific post-earthquake population, the sample size was calculated using the known-population formula. Therefore, the calculation was based on prevalence estimation rather than an expected effect size for VAS differences or regression analysis. This issue was considered when interpreting subgroup comparisons. The study included women who agreed to participate, were between 38 and 42 gestational weeks, had a singleton healthy fetus, were admitted in active labor, had regular uterine contractions, and were expected to give birth vaginally. Women were not enrolled if they had high-risk pregnancies, multiple pregnancies, gestational diabetes, fetal presentation anomalies, obstetric complications, irregular uterine contractions, pharmacological analgesia use before VAS assessment, or admission during the transition phase, because baseline pain assessment could not be performed. During the study period, 650 women admitted to the delivery unit for vaginal birth were screened for eligibility. Of these, 336 women were excluded before enrollment because they did not meet the inclusion criteria or met at least one exclusion criterion. The reasons for exclusion were high-risk pregnancy or obstetric complication (*n* = 54), admission during the transition phase before baseline VAS assessment could be performed (*n* = 42), pharmacological analgesia before the first VAS assessment (*n* = 48), irregular uterine contractions (*n* = 38), gestational diabetes or other pregnancy-related risk condition (*n* = 45), fetal presentation anomaly or multiple pregnancy (*n* = 32), and refusal to participate (*n* = 77). Finally, 314 eligible women were enrolled in the study. Participants enrolled during the active phase were followed prospectively throughout labor, and labor pain intensity was assessed using the VAS at 6 cm, 8 cm, and full cervical dilatation (10 cm). All enrolled women completed VAS assessments at all three measurement points, and no participant was excluded after enrollment because of incomplete VAS assessment, pharmacological analgesia during follow-up, or emergency obstetric intervention. Therefore, the final analysis included all 314 enrolled women. The participant-flow diagram is presented in Supplementary Figure S1.

### 2.3. Ethical Considerations

The study was conducted in accordance with the principles of the Declaration of Helsinki. Ethical approval was obtained from the Istanbul Atlas University Ethics Committee on 17 February 2025 with decision no. 02/44. Institutional permission was obtained from the relevant health authorities and Hatay Training and Research Hospital before data collection. Before data collection, all participants were informed verbally and in writing about the aim and scope of the study, the procedures to be performed, voluntary participation, confidentiality of data, and their right to withdraw from the study at any time. Written informed consent was obtained from all participants.

### 2.4. Setting and Time

The study data were collected in the delivery room of Hatay Training and Research Hospital, located in the Antakya district of Hatay. The delivery unit has eight LDRP rooms. The hospital serves a socio-culturally diverse population in Hatay and was therefore considered an appropriate setting for the study. Data were collected between February and June 2025.

### 2.5. Data Collection Instruments

Data were collected using a researcher-developed questionnaire form, the Ten-Item Personality Inventory, and the Visual Analog Scale. The researcher-developed questionnaire was used to obtain information on sociodemographic, obstetric, cultural, and post-disaster characteristics. The Ten-Item Personality Inventory was used to assess personality-related characteristics, and the Visual Analog Scale was used to quantify subjective labor pain intensity.

Questionnaire Form: The questionnaire was developed by the researchers based on the relevant literature and consisted of 52 items grouped into three sections. The first section comprised 15 items assessing sociodemographic characteristics, including age, educational level, duration of marriage, occupation, income status, hometown, religious belief, and ethnic origin. The second section comprised 12 items evaluating obstetric history, such as pregnancy history, number of pregnancies, previous birth history, stillbirths, previous birth history and mode, pregnancy follow-up, and education received regarding pregnancy and childbirth. The third section comprised 25 items focusing on cultural factors, including adherence to traditions, relationships with relatives and the opposite sex, attitudes toward male healthcare personnel, perceptions of childbirth and labor pain, factors influencing pain perception, and methods used to cope with pain [17,18,19]. Self-defined personality type was assessed using a single questionnaire item in which participants identified themselves as introverted or extroverted. This variable was analyzed separately from the TIPI Extraversion subscale. Official marriage status referred to whether the participant’s marriage was legally registered under civil law.

The cultural items were generated after reviewing previous studies on cultural beliefs, childbirth-related attitudes, labor pain perception, and sociocultural factors affecting women’s birth experiences. These items were designed to capture context-specific beliefs and attitudes relevant to childbirth in Hatay, including traditional rules, gender-related sensitivities during care, perceptions of touch, religious meaning attributed to labor pain, and culturally shaped coping behaviors. Because some items addressed sensitive issues, such as examination by male healthcare professionals and perceptions of touch, data were collected through face-to-face interviews in a respectful and non-judgmental manner. Participants were allowed to ask for clarification when needed, and explanations were provided without directing their responses.

The questionnaire was researcher-developed and was not intended to function as a standardized psychometric scale; therefore, no total cultural belief score was calculated. No formal pilot testing or psychometric validation was conducted; therefore, cultural items were analyzed individually as descriptive, exploratory, and context-specific variables. The full researcher-developed questionnaire is provided as Supplementary File S1 to improve transparency and reproducibility.

Post-disaster context was assessed using two proxy indicators: whether the participant had experienced the 2023 Türkiye earthquakes and current place of residence, categorized as home or container settlement. These variables were included to describe the post-earthquake context of the sample; however, they were not intended to capture the full severity or multidimensional burden of disaster exposure.

Ten-Item Personality Inventory (TIPI): Personality-related characteristics were assessed using the Ten-Item Personality Inventory, developed by Gosling et al. [20]. The scale consists of 10 items and measures five broad personality dimensions: Extraversion, Agreeableness, Conscientiousness, Emotional Stability, and Openness to Experience. Each dimension is assessed with two items on a seven-point Likert scale ranging from 1 = strongly disagree to 7 = strongly agree. The Turkish validity and reliability study was conducted by Atak [21]. In the present study, reliability coefficients were recalculated after verifying reverse coding and subscale assignment. Cronbach’s alpha coefficients were 0.849 for Extraversion, 0.167 for Agreeableness, 0.590 for Conscientiousness, 0.258 for Emotional Stability, and 0.339 for Openness to Experience. Because each TIPI dimension consists of two items, Spearman–Brown coefficients were also calculated as supplementary reliability indicators. The Spearman–Brown coefficients were 0.850 for Extraversion, 0.167 for Agreeableness, 0.591 for Conscientiousness, 0.259 for Emotional Stability, and 0.339 for Openness to Experience. Detailed inter-item correlations and reliability coefficients are presented in Supplementary Table S1. These results indicated high internal consistency for Extraversion, relatively better but still limited internal consistency for Conscientiousness, and low internal consistency for Agreeableness, Emotional Stability, and Openness to Experience. Therefore, personality-related findings, especially those involving low-reliability subscales, were interpreted cautiously and considered exploratory rather than confirmatory.

Visual Analog Scale (VAS): Labor pain intensity was assessed using the Visual Analog Scale (VAS). The VAS is a one-dimensional self-report scale used to measure subjective pain intensity. It consists of a 10-cm horizontal line, with one end representing “no pain” (0) and the other end representing “the worst pain imaginable” (10). Participants were asked to mark the point on the line that best reflected their pain intensity. The distance from the “no pain” end to the participant’s mark was measured and recorded as the pain score. Scores were interpreted as follows: 0 = no pain, 0.5–3.0 = mild pain, 3.5–6.5 = moderate pain, and 7.0–10.0 = severe pain [22].

### 2.6. Data Collection

The purpose of the study was explained to the pregnant women, and written informed consent was obtained from those who agreed to participate. Women who met the inclusion criteria were enrolled in the study. Data were collected through face-to-face interviews using the questionnaire form and the Ten-Item Personality Inventory. Monitoring began when the women were admitted to the delivery unit, and initial information regarding the birth process was obtained at that time. Labor pain intensity was assessed using the VAS at three time points: at 6 cm, 8 cm, and full cervical dilatation (10 cm).

### 2.7. Data Analysis

The data were analyzed using IBM SPSS Statistics version 25.0 (IBM Corp., Armonk, NY, USA). Descriptive statistics were presented as mean, standard deviation, minimum, and maximum values for continuous variables, and as frequencies and percentages for categorical variables. The normality of continuous variables was assessed using visual methods, including histograms and Q–Q plots, together with the Shapiro–Wilk test. For comparisons between two independent groups, the independent-samples t-test was used for normally distributed variables, while the Mann–Whitney U test was used for non-normally distributed variables. For comparisons among more than two groups, one-way analysis of variance (ANOVA) was used for normally distributed variables, and the Kruskal–Wallis H test was used for non-normally distributed variables. When significant differences were found, appropriate post-hoc analyses were performed to identify between-group differences. Because VAS pain scores were measured repeatedly in the same participants at 6 cm, 8 cm, and full cervical dilatation (10 cm), repeated-measures ANOVA was used to evaluate within-person changes in pain intensity across cervical dilatation points. This repeated-measures analysis was considered the primary analysis of pain progression. The assumption of sphericity was assessed using Mauchly’s test. When pairwise comparisons were performed, Bonferroni adjustment was applied. Effect size for the repeated-measures ANOVA was reported as partial eta squared (partial η^2^). Subgroup comparisons examining VAS scores and personality-related scores according to sociodemographic, obstetric, cultural, and post-disaster variables were considered exploratory and unadjusted. For exploratory unadjusted comparisons of VAS scores at full cervical dilatation (10 cm), Mann–Whitney U tests were used for two-category variables and Kruskal–Wallis tests were used for variables with three or more categories. Age was examined separately as a continuous variable using Spearman correlation. Effect size was reported as r for Mann–Whitney U tests and epsilon squared (ε^2^) for Kruskal–Wallis tests. For Supplementary Table S2, exploratory subgroup comparisons of TIPI subscale scores were conducted using nonparametric tests because TIPI subscale scores were derived from bounded two-item Likert-type scales and several subgroup sizes were unequal. Mann–Whitney U tests were used for two-group comparisons, and Kruskal–Wallis tests were used for comparisons involving three or more groups. Effect sizes were reported as r for Mann–Whitney U tests and epsilon squared (ε^2^) for Kruskal–Wallis tests. In addition, a multivariable linear regression model was conducted using VAS pain score at full cervical dilatation (10 cm) as the dependent variable. The model included age, education level, number of births, attendance at antenatal education, current place of residence, importance given to traditional rules, perception of touch, previous birth history and mode, Extraversion, and Conscientiousness. Regression coefficients, 95% confidence intervals, *p*-values, and variance inflation factors were reported. Because of the observational design, adjusted associations were interpreted cautiously and were not considered causal. Statistical significance was accepted at *p* < 0.05.

## 3. Results

A total of 314 women were included in the study. The mean age of the participants was 28.26 ± 7.57 years. Most participants were housewives (93%), and the most common education level was secondary school (29.9%), followed by high school (24.2%), illiteracy (22%), and primary school (14.6%). Most participants reported a moderate-income level (77.4%). Regarding proxy indicators of post-disaster context, 97.1% of the women had experienced the 2023 Türkiye earthquakes, and 51% were living in container settlements, while 49% were living at home. Most participants lived in nuclear families (75.2%). Regarding obstetric characteristics, 39.8% had two or three pregnancies, and 34.4% had no previous birth history. Among women with previous birth history, 55.3% had a previous spontaneous vaginal birth. Most pregnancies were planned (84.4%), and 90.4% of the women had attended antenatal follow-up visits. However, only 21.3% had received childbirth-related education during pregnancy, and 14% had attended antenatal education. In terms of self-defined personality, 71.3% of participants described themselves as introverted and 28.7% as extroverted (Table 1).

As shown in Table 2, almost half of the participants reported giving high importance to traditional rules (45.2%), while 47.8% gave moderate importance. Most women stated that they continued their previous traditions after coming to Hatay to the same extent as before (81.8%). Nearly half of the participants perceived being touched by someone as harassment (45.5%), and 42.4% reported that being touched by a male healthcare professional for examination would increase their pain. In relation to childbirth and labor pain, 45.9% of participants attributed religious meaning to labor pain, and 54.5% stated that labor pain was culturally described as something that must be experienced. Almost all participants reported praying to relieve labor pain (97.1%).

**Figure 1:**
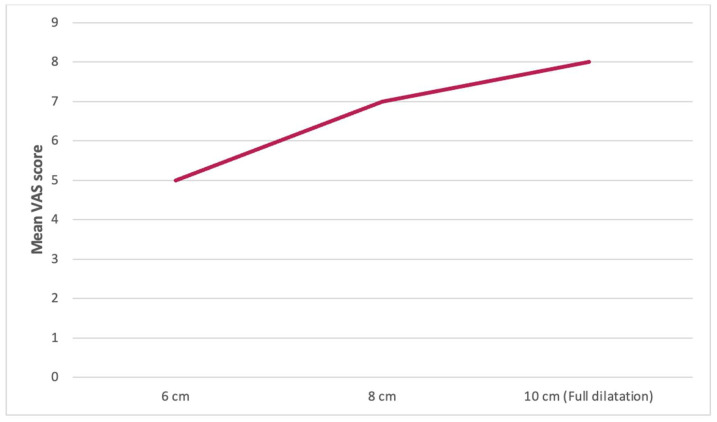
Progression of mean VAS pain scores across cervical dilatation points. The *x*-axis represents cervical dilatation points at 6 cm, 8 cm, and 10 cm; 10 cm indicates full cervical dilatation. The *y*-axis represents mean VAS pain scores. VAS scores increased significantly from 6 cm to 8 cm and from 8 cm to full cervical dilatation according to Bonferroni-adjusted pairwise comparisons, all p < 0.001. The corresponding 95% confidence intervals are presented in Table 3.

VAS pain scores showed a statistically significant progressive increase across cervical dilatation points. The mean VAS score was 5.04 ± 0.81 at 6 cm cervical dilatation, 7.01 ± 0.82 at 8 cm cervical dilatation, and 8.06 ± 0.93 at full cervical dilatation (10 cm). Mauchly’s test indicated that the assumption of sphericity was met, χ^2^(2) = 1.942, *p* = 0.379. Repeated-measures ANOVA demonstrated a significant within-person change in VAS scores across the three measurement points, F(2, 626) = 996.444, *p* < 0.001, partial η^2^ = 0.761. Bonferroni-adjusted pairwise comparisons showed that VAS scores increased significantly from 6 cm to 8 cm, from 8 cm to full cervical dilatation, and from 6 cm to full cervical dilatation, all *p* < 0.001. The mean VAS scores are presented in Table 3, and their progression across cervical dilatation points is illustrated in Figure 1.

The highest mean TIPI subscale score was observed for Agreeableness (4.17 ± 0.80), followed by Extraversion (4.12 ± 1.63). The lowest mean score was observed for Openness to Experience (3.3 ± 0.87). The mean VAS score at full cervical dilatation (10 cm) was 8.06 ± 0.93 (Table 4).

The reliability analysis showed that the internal consistency of the TIPI subscales varied considerably. Extraversion showed high internal consistency, and Conscientiousness showed relatively better but still limited internal consistency. In contrast, Agreeableness, Emotional Stability, and Openness to Experience showed low internal consistency. Therefore, personality-related findings, particularly those based on low-reliability subscales, were interpreted cautiously and considered exploratory rather than confirmatory.

Age was examined separately as a continuous variable using Spearman correlation. No statistically significant correlation was found between age and VAS pain score at full cervical dilatation (10 cm) (Spearman’s ρ = 0.021, *p* = 0.709, *n* = 314). Exploratory unadjusted subgroup comparisons of VAS pain scores at full cervical dilatation (10 cm) are presented in Table 5. VAS scores differed significantly according to education level, official marriage status, previous birth history and mode, attendance at antenatal education, and praying to relieve labor pain. In contrast, importance given to traditional rules, perception of being touched, self-defined personality type, current residence, and earthquake experience were not significantly associated with VAS scores in these exploratory unadjusted comparisons. Because these analyses were exploratory and unadjusted, the findings should not be interpreted as independent predictors. Effect sizes are also presented in Table 5 to support interpretation of the magnitude of group differences.

A multivariable linear regression model was conducted with VAS pain score at full cervical dilatation (10 cm) as the dependent variable. In response to the corrected reliability analysis, Extraversion and Conscientiousness were added to the adjusted model because these TIPI subscales showed the most acceptable reliability in the present sample. The overall model was statistically significant, F(13, 300) = 4.390, *p* < 0.001, and explained 16.0% of the variance in VAS scores (R^2^ = 0.160; adjusted R^2^ = 0.123). In the adjusted model, higher Extraversion was associated with lower VAS scores (B = −0.104, 95% CI: −0.187 to −0.020, *p* = 0.015), whereas Conscientiousness was not significantly associated with VAS scores (B = 0.077, 95% CI: −0.058 to 0.213, *p* = 0.263). Higher education level was associated with lower VAS scores (B = −0.193, 95% CI: −0.291 to −0.095, *p* < 0.001). Attendance at antenatal education (B = 0.530, 95% CI: 0.187 to 0.872, *p* = 0.003), greater importance given to traditional rules (B = 0.235, 95% CI: 0.037 to 0.432, *p* = 0.020), previous assisted vaginal/cesarean birth compared with no previous birth (B = −0.390, 95% CI: −0.740 to −0.040, *p* = 0.029), and current place of residence (B = 0.243, 95% CI: 0.006 to 0.481, *p* = 0.045) were also independently associated with VAS scores. Age, number of previous births, previous spontaneous vaginal birth, and touch perception categories were not significantly associated with VAS scores. No serious multicollinearity was observed, as all VIF values were below 5 (Table 6).

Exploratory subgroup comparisons of personality-related subscale scores according to selected sociodemographic, obstetric, and post-disaster characteristics are presented in Supplementary Table S2.

## 4. Discussion

This study examined the association of personality-related characteristics, cultural beliefs, obstetric characteristics, and proxy indicators of post-disaster context with labor pain perception among women giving birth in Hatay. The main finding was that VAS pain scores increased significantly across cervical dilatation points, indicating a clear within-person pain trajectory from 6 cm to full cervical dilatation (10 cm). In exploratory unadjusted comparisons, VAS scores at full cervical dilatation differed according to education level, official marriage status, previous birth history and mode, attendance at antenatal education, and praying to relieve labor pain. In the multivariable regression model, higher Extraversion and higher education level were associated with lower VAS scores, whereas attendance at antenatal education, greater importance given to traditional rules, previous assisted vaginal/cesarean birth, and current place of residence were independently associated with VAS scores. Conscientiousness and touch perception categories were not significantly associated with VAS scores in the adjusted model. These findings should be interpreted cautiously because subgroup comparisons were exploratory and unadjusted, the personality-related findings were limited by the low internal consistency of several TIPI subscales, and the observational design does not allow causal inference. Personality-related findings were interpreted cautiously because the corrected reliability analysis showed substantial variation across TIPI subscales. Extraversion showed high internal consistency, whereas Agreeableness, Emotional Stability, and Openness to Experience showed low internal consistency. Therefore, findings involving these lower-reliability subscales should be considered exploratory and should not be interpreted as confirmatory evidence of stable personality-related differences in labor pain perception.

In the present study, VAS pain scores increased progressively from 6 cm to 8 cm and full cervical dilatation, with the highest pain intensity reported at 10 cm full cervical dilatation. This finding is consistent with the physiological course of labor, as cervical dilatation, uterine contraction frequency, fetal descent, and pressure on pelvic structures intensify as labor progresses. Similar findings were reported by Buran et al. (2022), who found that VAS scores varied across the latent, active, and transition phases of labor and that pain was particularly prominent in the later stages of labor [23]. Likewise, Aslantaş et al. (2024) reported that labor pain became more pronounced as cervical dilatation progressed and that pain scores at advanced cervical dilatation were clinically meaningful for evaluating the effectiveness of intrapartum supportive interventions [24]. The similarity between these findings and our results is expected because labor pain is closely related to the biological progression of labor. However, the high pain scores observed in our study may also reflect the absence of pharmacological analgesia, as women who received analgesia were excluded from the study.

Education level was one of the most important variables associated with labor pain perception in this study. Illiterate women reported the highest VAS pain scores, whereas women with university or higher education had the lowest pain scores. This finding is compatible with the study by Huang et al. (2024), in which perceived labor pain intensity was negatively correlated with education level, family support, and childbirth self-efficacy [25]. The similarity may be explained by the role of education in improving access to health information, communication with healthcare professionals, awareness of the birth process, and use of coping strategies. Women with higher education may interpret labor sensations with less uncertainty and may feel more able to participate in decision-making during birth. In contrast, lower educational status may increase dependence on culturally transmitted narratives about pain, fear, and endurance, which may intensify pain perception. Therefore, the relationship between education and labor pain may not be purely cognitive; it may also reflect differences in empowerment, health literacy, and perceived control during childbirth.

Age was examined as a continuous variable and was not significantly correlated with VAS pain score at full cervical dilatation. Similarly, age was not significantly associated with VAS scores in the multivariable model. Therefore, age was not retained as a main factor associated with labor pain perception in this study. Although age may interact with obstetric history, psychological state, and social context, the present findings do not support a direct independent association between age and labor pain intensity.

Previous birth history and mode differed according to VAS scores in exploratory unadjusted comparisons; however, this association did not remain significant in the multivariable model. Therefore, this finding should be interpreted cautiously and should not be considered an independent predictor of labor pain perception. In the unadjusted analysis, women with a previous spontaneous vaginal birth reported higher VAS scores than those with a previous assisted vaginal birth or cesarean section. Although this may appear unexpected, previous birth experience does not always protect women from fear or pain. Huang et al. emphasized that previous traumatic or painful birth experiences may shape later fear of childbirth and pain expectations [25]. Similarly, Adler et al. reported that childbirth experience is influenced not only by mode of birth but also by operative delivery, complications, and women’s subjective evaluation of the birth process [26]. In this context, the unadjusted association observed in the present study may reflect previous pain memories, expectations about labor, or clustering with other obstetric and sociodemographic factors rather than an independent effect of previous birth history and mode.

Attendance at antenatal education was associated with higher VAS scores in our study. This finding differs from studies reporting that structured prenatal or childbirth preparation programs reduce labor pain, fear, and negative birth experiences. For example, Buran and Aksu found that hypnobirthing training significantly reduced VAS scores in the latent, active, and transition phases of labor [23]. Ucar and Golbasi also reported that an educational program based on cognitive behavioral techniques reduced fear of childbirth and positively affected the birth process [27]. However, our findings may be explained by several factors. First, antenatal education in our sample may not have been standardized or specifically focused on pain coping. Second, women who voluntarily attended education may have been more anxious, more concerned about birth, or more aware of pain-related risks before education. Third, the number of women attending antenatal education was low, which may have affected the stability of this comparison. Therefore, the higher VAS scores among women attending antenatal education should not be interpreted as evidence that antenatal education increases labor pain. Rather, this finding may reflect self-selection of women with greater fear, anxiety, or perceived need for support, as well as differences in the content, timing, and quality of antenatal education.

Interestingly, although attendance at antenatal education was associated with higher VAS pain scores, women who received childbirth-related education during follow-up had higher scores in several personality dimensions, including extraversion, agreeableness, conscientiousness, emotional stability, and openness to experience. This suggests that women with more adaptive interpersonal and self-regulatory traits may be more likely to seek, accept, or benefit from childbirth-related information. The literature supports the idea that education-based interventions are not uniform in their effects. Werner et al. found no significant difference in pain experience or epidural use after a brief self-hypnosis intervention compared with relaxation or usual care [28]. This shows that the effectiveness of education may depend on the content, duration, timing, cultural suitability, and women’s readiness to use the taught strategies. Therefore, in our study, antenatal education may have reflected women’s motivation and personality profile more than an effective pain-reduction intervention.

Exploratory personality-related scores differed according to several sociodemographic and obstetric variables. Women with higher education and those who were employed or self-employed had higher Extraversion, Conscientiousness, Emotional Stability, and Openness to Experience scores. These findings may suggest that social position, educational opportunities, and broader life experiences are related to the expression of personality-related characteristics in the childbirth context. In labor, personality-related characteristics such as Extraversion and Conscientiousness may be relevant to communication with healthcare professionals, asking questions, expressing needs, and using coping strategies. This interpretation is consistent with the study by Downe et al., which showed that women’s attitudes, beliefs, fear levels, and sense of control were linked to their experience of birth [29]. In other words, personality-related differences may be indirectly related to labor pain expression through perceived control, communication, expectations, and coping responses. However, these findings should be interpreted with caution. Although the TIPI was selected because of its brevity and feasibility in a delivery-room setting, the corrected reliability analysis showed substantial variation across subscales. Extraversion showed high internal consistency, and Conscientiousness showed relatively better but still limited internal consistency. In contrast, Agreeableness, Emotional Stability, and Openness to Experience showed low internal consistency. Therefore, findings involving the lower-reliability subscales, especially Emotional Stability and Openness to Experience, should be regarded as exploratory and hypothesis-generating rather than as confirmatory evidence that stable personality-related characteristics are independently associated with labor pain perception.

Cultural beliefs were also relevant to labor pain perception in this study. Although importance given to traditional rules did not reach statistical significance in exploratory unadjusted comparisons, it was positively associated with VAS scores in the multivariable regression model. This suggests that culturally shaped beliefs may be related to labor pain perception when considered together with sociodemographic and obstetric factors. Childbirth pain is not only a sensory experience but also a culturally interpreted event. In societies where labor pain is framed as necessary, meaningful, sacred, or morally valued, women may interpret pain as an expected part of motherhood or as something that should be endured. However, such meanings may also increase the emotional burden of pain, particularly when women feel that expressing pain is socially inappropriate. A study by Haines et al. found that women’s attitudes and beliefs about birth formed different profiles, including women who viewed birth as natural and manageable and women who were more fearful and concerned about pain, control, and safety [30]. Therefore, traditional beliefs may influence both the meaning attributed to labor pain and the acceptable ways of expressing it, but these findings should be interpreted cautiously because the cultural items were exploratory and context-specific.

Perception of being touched showed a clinically meaningful pattern in descriptive and exploratory analyses, although the exploratory unadjusted comparison did not reach statistical significance and touch perception categories were not independently associated with VAS scores in the adjusted model. VAS scores tended to be lower among women who perceived touch as interest/care, whereas scores were higher among women who perceived touch as affection, harassment, or depending on the person. Therefore, this pattern should be interpreted as exploratory rather than as evidence of an independent association. Nevertheless, the perceived meaning, context, predictability, safety, and acceptability of touch remain clinically important during intrapartum care. Although not specific to labor, Packheiser et al. showed that touch interventions may have physical and mental health benefits, but that the meaning, context, and acceptability of touch are crucial for its effect [31]. Because physical examination, positioning support, and close bodily interaction are common components of labor care, midwives should provide clear explanations, obtain consent, protect privacy, and use culturally sensitive communication before touching or examining women.

Religious coping was highly prevalent in this study, and almost all women reported praying to relieve labor pain. Women who reported praying had higher VAS scores than those who did not; however, this finding should be interpreted with particular caution because almost all participants reported praying and the comparison group was very small. Therefore, this result should not be interpreted as indicating that prayer increases pain. It may instead suggest that women experiencing more intense pain are more likely to use prayer as a culturally meaningful coping strategy. This interpretation is consistent with research showing that spiritual and religious coping may become more visible during uncontrollable, painful, or uncertain life events. In the childbirth context, prayer may provide meaning, endurance, emotional containment, and a sense of connection with God. Therefore, midwives should not interpret religious coping as a passive response; instead, they should recognize it as a culturally meaningful coping resource while also offering evidence-based pain management and emotional support.

Proxy indicators of post-disaster context were also considered in this study. In exploratory unadjusted comparisons, VAS pain scores did not differ significantly according to earthquake experience or current place of residence. However, in the adjusted model, current place of residence was independently associated with VAS scores, whereas earthquake experience was not. These findings should be interpreted cautiously for several reasons. First, 97.1% of the participants had experienced the earthquake, leaving very limited variability for comparing exposed and unexposed women. Second, current place of residence was only a rough proxy for post-disaster living context and did not capture the severity of displacement, housing damage, perceived safety, privacy, social support, healthcare access, or trauma-related symptoms. Therefore, the present findings should not be interpreted as a comprehensive assessment of disaster burden or as evidence that disaster-related conditions are unrelated to labor pain. Rather, they indicate that more detailed indicators of post-disaster living conditions are needed in future studies. In supplementary exploratory analyses, current place of residence was also associated with Emotional Stability; however, this finding should be interpreted with caution because the Emotional Stability subscale showed low internal consistency in the corrected reliability analysis. Recent evidence from Türkiye shows that pregnant women exposed to earthquakes may experience psychological trauma, including emotional, behavioral, cognitive, and sleep-related symptoms [32]. Similarly, qualitative research with postpartum women after the 2023 earthquakes showed that women experienced fear, anxiety, hopelessness, exhaustion, and difficulties related to pregnancy, birth, and postpartum care [33]. Taken together, these findings suggest that future studies should assess post-disaster childbirth experiences using more comprehensive indicators, including trauma-related symptoms, perceived safety, housing conditions, privacy, social support, and access to reproductive healthcare.

The finding that earthquake experience itself was not associated with VAS pain should also be interpreted with caution. Since 97.1% of the participants had experienced the earthquake, the study had very limited contrast between exposed and non-exposed women. For this reason, current living conditions, emotional stability, and access to antenatal support may be more meaningful indicators than the binary variable of earthquake exposure. This interpretation is supported by research on individualized care provided to pregnant women in earthquake-affected living spaces, which emphasizes that disaster conditions increase the need for qualified prenatal care, safe childbirth environments, psychosocial support, and midwifery counseling [34]. Therefore, in future studies, earthquake exposure should be measured with more detailed indicators such as loss of relatives, housing damage, displacement duration, perceived safety, social support, and post-traumatic stress symptoms.

Another important finding was that planned pregnancy was associated with agreeableness, conscientiousness, and openness to experience, but not with VAS pain scores. This suggests that pregnancy planning may be more closely related to women’s psychosocial preparedness and personality profile than to the sensory intensity of labor pain itself. Women with planned pregnancies may be more prepared for motherhood, more likely to seek antenatal care, and more open to pregnancy-related information. However, once active labor begins, physiological intensity, cervical dilatation, previous birth experience, cultural meaning, and immediate support may become more influential in determining VAS pain scores than pregnancy planning. This distinction is important because psychosocial preparedness may shape the quality of the birth experience even when it does not directly reduce pain intensity. The repeated-measures analysis strengthened this finding by demonstrating a significant within-person increase in VAS scores across the three cervical dilatation points.

The place of antenatal follow-up was associated with extraversion and agreeableness, but not with VAS pain scores. Women followed in family health centers or private hospitals had higher extraversion and agreeableness scores than women followed in hospitals. This may reflect differences in care-seeking behavior, communication style, socioeconomic status, or perceived accessibility of services. Extraverted and agreeable women may be more comfortable interacting with healthcare professionals and may prefer or access settings where communication is more personalized. However, the lack of association with VAS suggests that follow-up location alone is not sufficient to reduce labor pain unless antenatal care includes effective pain-coping education, psychosocial preparation, and individualized birth planning.

Overall, the findings of this study support the view that labor pain perception is multidimensional. Biological progression of labor explains the increase in pain intensity across cervical dilatation, but individual characteristics, education, previous birth experience, cultural meanings, touch perception, religious coping, and post-disaster context influence how pain is interpreted and expressed. Intervention studies also show that supportive strategies such as hypnobirthing, birth ball exercise, cognitive behavioral education, and other non-pharmacological approaches may reduce pain or improve birth experience when they are structured and appropriately delivered [23,27,33]. However, the mixed findings regarding antenatal education and self-hypnosis indicate that not all educational interventions are equally effective [28]. Therefore, midwifery care should not rely only on standard education but should assess women’s literacy level, cultural beliefs, previous birth memories, personality-related coping tendencies, privacy needs, and disaster-related vulnerabilities.

The main strength of this study is the prospective assessment of labor pain at three defined cervical dilatation points in a culturally diverse and earthquake-affected region. In addition, evaluating cultural attitudes, obstetric characteristics, personality-related characteristics, and proxy indicators of post-disaster context together provides a broader perspective on factors associated with labor pain perception. However, several limitations should be considered. First, the descriptive observational design prevents causal interpretation. Second, the sample size was calculated using a known-population formula rather than an expected effect size for VAS differences or multivariable analysis; therefore, subgroup and exploratory comparisons should be interpreted cautiously. Third, the findings mainly apply to women with uncomplicated vaginal births without pharmacological analgesia and should not be generalized to all laboring women in Hatay. Fourth, nearly all participants had experienced the earthquake, which limited the variability needed to examine the association between earthquake exposure and labor pain perception. Moreover, earthquake-related experiences were assessed using limited proxy indicators, such as earthquake experience and current place of residence, rather than detailed measures of displacement, loss, housing damage, perceived safety, social support, healthcare access, or post-traumatic stress symptoms. Therefore, this study should not be considered a comprehensive evaluation of disaster burden or its independent association with labor pain. Fifth, some subgroups, such as women who did not pray and women who were employed, were small, which may reduce the stability of subgroup comparisons. Sixth, the TIPI was used because it is brief and practical for use in a delivery-room setting; however, its two-item subscale structure limits the estimation of internal consistency. In the corrected reliability analysis, Extraversion showed high internal consistency and Conscientiousness showed relatively better but still limited internal consistency, whereas Agreeableness, Emotional Stability, and Openness to Experience showed low internal consistency. Therefore, findings involving the lower-reliability TIPI subscales should be interpreted with substantial caution, and personality-related findings should be considered exploratory and hypothesis-generating rather than confirmatory. Future studies should use more comprehensive personality measures when the primary aim is to examine personality-related differences in labor pain perception. Finally, cultural beliefs and childbirth-related attitudes were assessed using a researcher-developed questionnaire. Although the items were based on the relevant literature and were designed to reflect the sociocultural context of Hatay, the questionnaire did not undergo formal psychometric validation. Accordingly, cultural findings should be interpreted as exploratory and context-specific, with limited comparability to studies using validated cultural belief measures.

## 5. Conclusions

Labor pain intensity increased progressively from 6 cm cervical dilatation to full cervical dilatation (10 cm), confirming the clinical importance of assessing pain trajectory during labor rather than relying on a single measurement point. Labor pain perception was associated with selected sociodemographic, obstetric, cultural, personality-related, and contextual characteristics. In the adjusted model, higher Extraversion and higher education level were associated with lower VAS scores, whereas attendance at antenatal education, greater importance given to traditional rules, previous assisted vaginal/cesarean birth, and current place of residence were independently associated with VAS scores at full cervical dilatation. Touch perception categories were not independently associated with VAS scores in the adjusted model. These findings support the importance of individualized, culturally sensitive, and trauma-informed midwifery care, particularly in disaster-affected regions.

Personality-related findings should be interpreted cautiously. The corrected reliability analysis showed high internal consistency for Extraversion and relatively better but still limited internal consistency for Conscientiousness, whereas Agreeableness, Emotional Stability, and Openness to Experience showed low internal consistency. Therefore, findings involving the lower-reliability TIPI subscales should be considered exploratory and hypothesis-generating rather than confirmatory evidence that stable personality-related characteristics independently explain labor pain perception. Similarly, disaster-related findings should be interpreted cautiously because post-disaster context was assessed using only limited proxy indicators, namely earthquake experience and current place of residence. Therefore, the present study should not be interpreted as providing a comprehensive assessment of disaster burden or its independent association with labor pain. Because of the observational design, causal inference cannot be made. Future studies in disaster-affected regions should include more detailed measures of displacement, trauma exposure, housing conditions, perceived safety, social support, healthcare access, and post-traumatic stress symptoms.

## Figures and Tables

**Table 1:** Sociodemographic, obstetric and post-disaster characteristics of participants (*n* = 314).

Variable	Category	Mean ± SD	
Age		28.26 ± 7.57	
	Category	*n*	%
Education level	Illiterate	69	22
	Primary school	46	14.6
	Secondary school	94	29.9
	High school	76	24.2
	University or higher	29	9.2
Occupation	Housewife	292	93
	Employed/self-employed	22	7
Income status	Poor	41	13.1
	Moderate	243	77.4
	Good	30	9.6
Experienced earthquake	Yes	305	97.1
	No	9	2.9
Current living place	Container settlement	160	51
	Home	154	49
Family type	Nuclear family	236	75.2
	Extended family	78	24.8
Official marriage status	No	62	19.7
	Yes	252	80.3
Number of pregnancies	1	101	32.2
	2–3	125	39.8
	≥4	88	28
Previous birth history	No previous birth	108	34.4
	Previous birth	206	65.6
Previous birth history and mode *	Spontaneous vaginal birth	114	55.3
	Assisted vaginal birth/cesarean section	92	44.7
Planned pregnancy	Yes	265	84.4
	No	49	15.6
Antenatal follow-up	Yes	284	90.4
	No	30	9.6
Childbirth-related education during follow-up	Yes	67	21.3
	No	247	78.7
Attendance at antenatal education	Yes	44	14
	No	270	86
Self-defined personality	Extroverted	90	28.7
	Introvert	224	71.3

* Among women who had previously given birth. *n* = Number, % = Percent.

**Table 2:** Selected cultural beliefs and attitudes related to childbirth and labor pain.

Variable	Category	*n*	%
Importance given to traditional rules	Low	22	7
	Moderate	150	47.8
	High	142	45.2
Continuation of previous traditions after coming to Hatay	To a lesser extent	40	12.7
	Same as before	257	81.8
	Not continued	17	5.4
Discomfort with male healthcare personnel during birth	Yes	137	43.6
	No	177	56.4
Perception of being touched by someone	Affection	41	13.1
	Interest/care	31	9.9
	Harassment	143	45.5
	Depends on the person	99	31.5
Belief that examination by a male healthcare professional increases pain	Yes	133	42.4
	No	181	57.6
Being accompanied in the labor pain room increases pain	Yes	212	67.5
	No	102	32.5
Cultural belief about labor pain	Something that must be experienced	171	54.5
	Unbearable	77	24.5
	Sacred	66	21
Religious meaning attributed to labor pain	Yes	144	45.9
	No	170	54.1
Praying to relieve labor pain	Yes	305	97.1
	No	9	2.9
Desire to have a religious object nearby	No	179	57
	Qur’an	114	36.3
	Amulet/cevşen	21	6.7
Desire to scream during labor pain	Yes	269	85.7
	No	45	14.3

**Table 3:** Mean VAS pain scores across cervical dilatation points.

Cervical Dilatation Point	Mean ± SD	SE	95% CI
6 cm	5.04 ± 0.81	0.046	4.948–5.129
8 cm	7.01 ± 0.82	0.046	6.915–7.098
Full cervical dilatation (10 cm)	8.06 ± 0.93	0.053	7.957–8.164

Note. Repeated-measures ANOVA showed a significant increase in VAS pain scores across cervical dilatation points, F(2, 626) = 996.444, *p* < 0.001, partial η^2^ = 0.761. Mauchly’s test indicated that the assumption of sphericity was met, χ^2^(2) = 1.942, *p* = 0.379. Bonferroni-adjusted pairwise comparisons showed significant differences between all measurement points (all *p* < 0.001).

**Table 4:** Mean TIPI subscale scores and labor pain perception.

Variable	Mean ± SD	Minimum	Maximum
Extraversion	4.12 ± 1.63	1	7
Agreeableness	4.17 ± 0.80	2	7
Conscientiousness	3.93 ± 0.98	1	7
Emotional Stability	3.8 ± 0.77	1	7
Openness to Experience	3.3 ± 0.87	1	6.5
VAS at full cervical dilatation (10 cm)	8.06 ± 0.93	6	10

**Table 5:** Exploratory unadjusted comparisons of VAS pain scores at full cervical dilatation (10 cm).

Variable	Category	*n*	VAS Mean ± SD	Test	*p*	Effect Size
Education level	Illiterate	69	8.45 ± 0.65	KW = 23.878	<0.001	ε^2^ = 0.064
	Primary school	46	8 ± 1.15			
	Secondary school	94	8.01 ± 0.86			
	High school	76	7.72 ± 0.87			
	University or higher	29	8.28 ± 1.13			
Official marriage status	No	62	8.34 ± 1.10	U = 5952.500	0.002	r = 0.174
	Yes	252	7.99 ± 0.87			
Previous birth history and mode	No previous birth	108	8.13 ± 0.81	KW = 10.173	0.006	ε^2^ = 0.026
	Spontaneous vaginal birth	114	8.23 ± 0.81			
	Cesarean/assisted birth	92	7.77 ± 1.13			
Attendance at antenatal education	No	270	8.01 ± 0.94	U = 4690.500	0.018	r = 0.134
	Yes	44	8.36 ± 0.81			
Importance given to traditional rules	Low	22	7.91 ± 0.97	KW = 4.980	0.083	ε^2^ = 0.010
	Moderate	150	7.95 ± 0.98			
	High	142	8.20 ± 0.86			
Perception of being touched	Affection	41	8.10 ± 1.09	KW = 7.385	0.061	ε^2^ = 0.014
	Interest/care	31	7.61 ± 0.99			
	Harassment	143	8.11 ± 0.92			
	Depends on the person	99	8.11 ± 0.83			
Praying to relieve labor pain	No	9	7.11 ± 0.93	U = 663.500	0.005	r = 0.158
	Yes	305	8.09 ± 0.92			
Self-defined personality	Extroverted	90	7.92 ± 1.12	U = 9009.500	0.119	r = 0.088
	Introverted	224	8.12 ± 0.84			
Current residence	Container settlement	160	8.03 ± 0.99	U = 11,835.000	0.523	r = 0.036
	Home	154	8.09 ± 0.87			
Experienced earthquake	No	9	8.33 ± 0.87	U = 1114.500	0.308	r = 0.058
	Yes	305	8.05 ± 0.93			

Note. VAS: Visual Analog Scale; SD: standard deviation; KW: Kruskal–Wallis test; U: Mann–Whitney U test. Effect size was reported as r for Mann–Whitney U tests and epsilon squared (ε^2^) for Kruskal–Wallis tests. These analyses were exploratory and unadjusted; therefore, *p*-values should be interpreted cautiously.

**Table 6:** Multivariable linear regression analysis of factors associated with VAS pain scores at full cervical dilatation (10 cm).

Variable	B	SE	β	95% CI for B	*p*	VIF
Extraversion	−0.104	0.042	−0.158	−0.187 to −0.020	0.015	1.475
Conscientiousness	0.077	0.069	0.071	−0.058 to 0.213	0.263	1.422
Age	−0.005	0.011	−0.035	−0.026 to 0.016	0.638	2.007
Education level	−0.193	0.050	−0.228	−0.291 to −0.095	<0.001	1.234
Number of previous births	0.063	0.060	0.090	−0.055 to 0.181	0.297	2.646
Attendance at antenatal education	0.530	0.174	0.171	0.187 to 0.872	0.003	1.126
Touch perception: affection	0.337	0.251	0.106	−0.157 to 0.831	0.181	2.212
Touch perception: harassment	0.140	0.209	0.065	−0.270 to 0.551	0.502	3.338
Touch perception depends on person	0.366	0.216	0.158	−0.058 to 0.791	0.090	3.100
Previous spontaneous vaginal birth	−0.306	0.180	−0.137	−0.660 to 0.049	0.091	2.320
Previous assisted vaginal/cesarean birth	−0.390	0.178	−0.165	−0.740 to −0.040	0.029	2.023
Importance given to traditional rules	0.235	0.100	0.134	0.037 to 0.432	0.020	1.169
Current place of residence	0.243	0.121	0.113	0.006 to 0.481	0.045	1.125

Note. Dependent variable: VAS pain score at full cervical dilatation (10 cm). Model fit: F(13, 300) = 4.390, *p* < 0.001; R^2^ = 0.160; adjusted R^2^ = 0.123. Reference categories were interest/care for touch perception and no previous birth for previous birth history and mode. VAS: Visual Analog Scale; CI: confidence interval; VIF: variance inflation factor.

## Data Availability

The data presented in this study are available on request from the corresponding author due to privacy and ethical restrictions, as the dataset contains sensitive participant information and was collected from human participants under informed consent conditions.

## Supplementary Materials

The following supporting information can be downloaded at: https://www.mdpi.com/article/10.3390/healthcare14131827/s1, File S1: Researcher-developed questionnaire form; Figure S1: Participant-flow diagram; Table S1: Reliability coefficients for TIPI two-item subscales; Table S2: Exploratory comparison of personality-related subscale scores according to selected sociodemographic, obstetric, and post-disaster characteristics.

## Abbreviations

The following abbreviations are used in this manuscript:

VAS	Visual Analog Scale
TIPI	Ten-Item Personality Inventory
LDRP	Labor, Delivery, Recovery, and Postpartum
SD	Standard Deviation
ANOVA	Analysis of Variance
