# Supplementary material for: Personality-Related Characteristics, Cultural Beliefs, and Labor Pain Perception After the 2023 Türkiye Earthquakes: A Prospective Study in Hatay
Source: Healthcare (Basel). 2026 Jun 23;14(13):1827. doi: 10.3390/healthcare14131827 (PMC13362373; doi:10.3390/healthcare14131827)
Supplement: Supplementary file 1 [file healthcare-14-01827-s001.zip › healthcare-4360334-supplementary/Supplementary File S1 Researcher developed questionnaire.pdf]

Supplementary File 1. Questionnaire Form on the Effect of Personality Characteristics and Cultural Structure on Labor Pain

SECTION ONE

A) Sociodemographic Information
1. How old are you? ....
2. Duration of marriage? ....
3. Educational level:
(1) Illiterate (2) Primary school (3) Secondary school (4) High school (5) University
4. What is your occupation?
(1) Housewife
(2) Worker
(3) Civil servant
(4) Self-employed
(5) Other: ....
5. Income status:
(1) Poor (2) Moderate (3) Good
6. What is your hometown? ....
7. How long have you been living here? ....
8. Did you experience the Hatay earthquake?
(1) Yes (2) No
9. Where do you live?
(1) Container settlement (2) Home
10. Which sect of Islam do you belong to?
(1) Sunni (2) Alevi
11. What is your ethnic origin?
(1) Turkish
(2) Kurdish
(3) Arab
(4) Other: ....
12. Whom do you live with?
(1) Nuclear family (2) Extended family
13. Do you have a consanguineous marriage with your spouse?
(1) Yes (2) No
14. If yes, what is your kinship relationship? ....
15. Do you have an official civil marriage?
(1) Yes (2) No

SECTION TWO

B) Obstetric History
16. Number of pregnancies: ....
17. Number of births: ....
18. Number of stillbirths: ....

19. Is this your first birth?
(1) Yes (2) No If no, previous mode of birth: (1) Spontaneous vaginal birth (2) Assisted vaginal birth (3) Cesarean section

20. Number of spontaneous/induced abortions: ....

21. Was this pregnancy planned/wanted?
(1) Yes (2) No

22. Did you attend pregnancy check-ups?
(1) Yes (2) No

23. If yes, where did you attend check-ups?

(1) Health house/family health center

(2) Hospital

(3) Private physician's office

(4) Private clinic

(5) Other: ....

24. Who performed the antenatal check-ups?

(1) Midwife

(2) Nurse

(3) Physician

(4) Other: ....

25. Did you receive education about childbirth during antenatal check-ups?
(1) Yes (2) No

26. Who provided the education?

(1) Midwife

(2) Nurse

(3) Physician

(4) Other: ....

27. Did you attend any antenatal education program?
(1) Yes (2) No

SECTION THREE

C) Cultural Factors
28. Do you give importance to traditional rules?
(1) I do not give much importance (2) I give moderate importance (3) I give great importance

29. After coming to Hatay, do you continue your previous traditions?

(1) I continue them to a lesser extent

(2) I continue them in the same way

(3) I do not continue them

(4) Other: ....

30. Does the presence of male healthcare personnel during birth make you uncomfortable?
(1) Yes (2) No

31. Which language do you use for communication within the family?

(1) Turkish

(2) Arabic

(3) Kurdish

(4) Other: ....

32. How do you perceive someone touching you?

(1) Affection

(2) Interest/care

(3) Harassment

(4) Other: ....

33. How would you feel about being touched by a male healthcare professional for examination purposes? (You may mark more than one option).

(1) I would feel embarrassed

(2) It is a disgusting situation

(3) It is a sin

(4) It is against traditions

(5) It is a normal situation

(6) My spouse would not approve

34. Would being touched by a male healthcare professional for examination purposes increase your pain?
(1) Yes (2) No

35. Does not being alone in the labor pain room, for example if the environment is crowded, increase your pain?
(1) Yes (2) No

36. In which situations do you raise your voice the most?

(1) When I am happy

(2) When I am tense

(3) When I am excited

(4) When I am afraid

(5) When I am in pain

(6) Other: ....

37. In which situations do you prefer to remain silent?

(1) Sadness

(2) Respect

(3) Illness

(4) Fear

(5) Anxiety

(6) Other: ....

38. How would you describe yourself?
(1) Introverted (2) Extroverted

39. How is pregnancy perceived in your culture?

(1) A normal condition

(2) A state of illness

(3) Other: ....

40. What is the meaning of childbirth? ....

41. What do you think is the meaning of labor pain?

(1) A normal condition

(2) An abnormal condition

(3) Something that must be experienced

(4) A woman cannot be a mother without experiencing labor pain
(5) Something that increases a woman's value
(6) A maturing experience for women
(7) Something that increases the baby's value
(8) Other: ....
42. What has been said in your culture, for example by family elders, about labor pain?
(1) Something that must be experienced
(2) An unbearable condition
(3) A sacred condition
(4) Other: ....
43. Who do you think experiences labor pain more intensely?
(1) People who are sinful
(2) People who are unbelievers
(3) People who are disrespectful toward family elders
(4) People who do not pay attention to cleanliness
(5) People affected by the evil eye
(6) Other: ....
44. Does labor pain have a religious meaning?
(1) Yes (2) No
45. If yes, what is it? ....
46. Do you pray to relieve labor pain?
(1) Yes (2) No
47. Should a woman shout or make noise during labor pain?
(1) Yes (2) No
48. Do you feel the desire to shout during labor pain?
(1) Yes (2) No
49. What might be the reason why a woman cannot shout during birth?
(1) Embarrassment
(2) Shyness toward healthcare personnel
(3) Shyness toward other pregnant women
(4) Other: ....
50. What have you heard from others about labor pain? ....
51. Would you like to have a religious object such as the Qur'an, an amulet, or cevşen with you?
(1) No
(2) Qur'an
(3) Amulet
(4) Cevşen
(5) Other: ....
52. What does labor pain make you feel? (You may mark more than one option).
(1) Self-confidence
(2) Sense of motherhood
(3) Achievement of a sacred duty
(4) Pain
(5) Weakness

(6) Loss of control / inability to control myself
(7) Fragility
(8) Other: ....
