# Supplementary material for: Personality-Related Characteristics, Cultural Beliefs, and Labor Pain Perception After the 2023 Türkiye Earthquakes: A Prospective Study in Hatay
Source: Healthcare (Basel). 2026 Jun 23;14(13):1827. doi: 10.3390/healthcare14131827 (PMC13362373; doi:10.3390/healthcare14131827)
Supplement: Supplementary file 1 [file healthcare-14-01827-s001.zip › healthcare-4360334-supplementary/Supplementary Table S1. Reliability coefficients for TIPI two-item subscales.pdf]

Supplementary Table S1. Reliability coefficients for TIPI two-item subscales

Subscale	Pearson r	Cronbach's alpha	Cronbach's alpha based on standardized items / Spearman–Brown coefficient	N
**Extraversion**	0.740	0.849	0.850	314
**Agreeableness**	0.091	0.167	0.167	314
**Conscientiousness**	0.420	0.590	0.591	314
**Emotional Stability**	0.149	0.258	0.259	314
**Openness to Experience**	0.204	0.339	0.339	314

Note. Reliability coefficients were recalculated after verifying reverse coding and subscale assignment. Pearson r refers to the inter-item correlation for each two-item TIPI subscale. Because each TIPI subscale includes two items, Cronbach's alpha based on standardized items is mathematically equivalent to the Spearman–Brown coefficient. Higher values indicate stronger internal consistency.
