# Supplementary material for: Personality-Related Characteristics, Cultural Beliefs, and Labor Pain Perception After the 2023 Türkiye Earthquakes: A Prospective Study in Hatay
Source: Healthcare (Basel). 2026 Jun 23;14(13):1827. doi: 10.3390/healthcare14131827 (PMC13362373; doi:10.3390/healthcare14131827)
Supplement: Supplementary file 1 [file healthcare-14-01827-s001.zip › healthcare-4360334-supplementary/Supplementary Table S2. Exploratory comparison of personality-related subscale scores according to selected sociodemographic, ob.pdf]

Supplementary Table S2. Exploratory comparison of personality-related subscale scores according to selected sociodemographic, obstetric, and post-disaster characteristics.

Variable	Category	Extraversion Mean ± SD	Agreeableness Mean ± SD	Conscientiousness Mean ± SD	Emotional Stability Mean ± SD	Openness Mean ± SD
**Education level**	**Illiterate**	3.70 ± 1.06	4.03 ± 0.74	3.75 ± 0.86	3.72 ± 0.80	3.39 ± 0.73
	**Primary school**	3.90 ± 1.36	4.29 ± 0.80	3.76 ± 0.71	3.75 ± 0.80	3.26 ± 0.80
	**Secondary school**	4.27 ± 1.70	4.22 ± 0.85	4.03 ± 0.92	3.82 ± 0.64	3.37 ± 0.87
	**High school**	4.02 ± 1.98	4.11 ± 0.79	3.82 ± 1.01	3.70 ± 0.75	3.02 ± 0.86
	**University or higher**	5.33 ± 1.36	4.34 ± 0.86	4.60 ± 1.42	4.31 ± 0.99	3.71 ± 1.13
	**Test / p / effect size**	KW = 25.796; $p < 0.001$; $\varepsilon^2 = 0.071$	KW = 3.512; p = 0.476; $\varepsilon^2 = 0.000$	KW = 7.036; p = 0.134; $\varepsilon^2 = 0.010$	KW = 11.629; p = 0.020; $\varepsilon^2 = 0.025$	KW = 17.984; p = 0.001; $\varepsilon^2 = 0.045$
**Occupation**	**Housewife**	4.00 ± 1.58	4.16 ± 0.81	3.87 ± 0.90	3.76 ± 0.73	3.26 ± 0.83
	**Employed/self-employed**	5.80 ± 1.40	4.32 ± 0.76	4.77 ± 1.59	4.45 ± 1.06	3.89 ± 1.21
	**Test / p / effect size**	U = 1207.500; $p < 0.001$; r = 0.277	U = 2788.000; p = 0.284; r = 0.061	U = 2426.000; p = 0.050; r = 0.110	U = 1908.000; $p < 0.001$; r = 0.187	U = 2406.000; p = 0.045; r = 0.113
**Income status**	**Poor**	4.29 ± 2.01	4.34 ± 0.73	4.09 ± 0.92	3.94 ± 0.85	3.41 ± 0.96
	**Moderate**	3.98 ± 1.56	4.13 ± 0.85	3.87 ± 0.99	3.74 ± 0.78	3.27 ± 0.89
	**Good**	5.07 ± 1.32	4.25 ± 0.49	4.22 ± 1.01	4.17 ± 0.55	3.43 ± 0.58
	**Test / p / effect size**	KW = 11.756; p = 0.003; $\varepsilon^2 = 0.031$	KW = 3.322; p = 0.190; $\varepsilon^2 = 0.004$	KW = 3.603; p = 0.165; $\varepsilon^2 = 0.005$	KW = 10.123; p = 0.006; $\varepsilon^2 = 0.026$	KW = 1.028; p = 0.598; $\varepsilon^2 = 0.000$
**Current place of residence**	**Container settlement**	3.99 ± 1.67	4.23 ± 0.64	4.04 ± 0.97	3.89 ± 0.77	3.37 ± 0.85
	**Home**	4.27 ± 1.58	4.12 ± 0.95	3.82 ± 0.99	3.72 ± 0.78	3.24 ± 0.90
	**Test / p / effect size**	U = 11201.500; p = 0.162; r = 0.079	U = 10722.000; p = 0.039; r = 0.116	U = 10248.000; p = 0.008; r = 0.149	U = 10666.500; p = 0.032; r = 0.121	U = 10847.500; p = 0.061; r = 0.106
**Family type**	**Nuclear family**	4.16 ± 1.64	4.16 ± 0.82	3.98 ± 0.97	3.86 ± 0.77	3.31 ± 0.89
	**Extended family**	4.04 ± 1.61	4.20 ± 0.79	3.78 ± 1.02	3.63 ± 0.80	3.30 ± 0.83
	**Test / p / effect size**	U = 8956.000; p = 0.720; r = 0.020	U = 9145.500; p = 0.930; r = 0.005	U = 8236.000; p = 0.155; r = 0.080	U = 7822.500; p = 0.038; r = 0.117	U = 9061.000; p = 0.834; r = 0.012
**Consanguineous marriage**	**No**	4.14 ± 1.70	4.19 ± 0.80	3.97 ± 1.00	3.85 ± 0.79	3.31 ± 0.92

	**Yes**	4.09 ± 1.30	4.11 ± 0.84	3.76 ± 0.89	3.58 ± 0.69	3.28 ± 0.61
	**Test / p / effect size**	U = 6829.500; p = 0.630; r = 0.027	U = 6825.000; p = 0.613; r = 0.028	U = 6201.500; p = 0.124; r = 0.087	U = 5553.500; p = 0.007; r = 0.151	U = 6994.500; p = 0.831; r = 0.012
**Official marriage status**	**No**	3.85 ± 1.20	4.00 ± 0.69	3.77 ± 1.19	3.56 ± 1.01	3.28 ± 0.93
	**Yes**	4.20 ± 1.72	4.21 ± 0.83	3.97 ± 0.93	3.87 ± 0.70	3.31 ± 0.86
	**Test / p / effect size**	U = 6754.500; p = 0.097; r = 0.094	U = 6974.500; p = 0.174; r = 0.077	U = 7197.500; p = 0.327; r = 0.055	U = 6061.500; p = 0.004; r = 0.161	U = 7714.500; p = 0.876; r = 0.009
**Previous birth history and mode**	**No previous birth**	4.19 ± 1.79	4.26 ± 0.77	3.89 ± 1.00	3.78 ± 0.80	3.40 ± 0.93
	**Previous spontaneous vaginal birth**	3.90 ± 1.42	4.14 ± 0.74	3.88 ± 0.96	3.77 ± 0.89	3.27 ± 0.88
	**Previous assisted vaginal/cesarean birth**	4.33 ± 1.66	4.10 ± 0.93	4.04 ± 1.00	3.88 ± 0.58	3.23 ± 0.79
	**Test / p / effect size**	KW = 4.520; p = 0.104; ε^2 = 0.008	KW = 2.293; p = 0.318; ε^2 = 0.001	KW = 0.389; p = 0.823; ε^2 = 0.000	KW = 1.603; p = 0.449; ε^2 = 0.000	KW = 3.617; p = 0.164; ε^2 = 0.005
**Planned pregnancy**	**No**	4.42 ± 1.76	3.90 ± 0.95	3.70 ± 0.94	3.91 ± 1.05	3.10 ± 1.08
	**Yes**	4.07 ± 1.60	4.22 ± 0.77	3.97 ± 0.99	3.78 ± 0.72	3.34 ± 0.83
	**Test / p / effect size**	U = 5650.500; p = 0.147; r = 0.082	U = 4874.500; p = 0.004; r = 0.162	U = 5240.500; p = 0.028; r = 0.124	U = 5748.500; p = 0.183; r = 0.075	U = 5336.500; p = 0.043; r = 0.114
**Place of antenatal follow-up**	**Hospital**	4.07 ± 1.58	4.15 ± 0.78	3.93 ± 0.87	3.85 ± 0.65	3.29 ± 0.76
	**Family health center/private clinic/private physician**	4.98 ± 1.85	4.58 ± 1.00	4.27 ± 1.13	3.62 ± 0.83	3.67 ± 1.19
	**Test / p / effect size**	U = 2303.000; p = 0.008; r = 0.157	U = 2681.000; p = 0.080; r = 0.104	U = 2665.500; p = 0.077; r = 0.105	U = 2680.000; p = 0.078; r = 0.105	U = 2999.500; p = 0.363; r = 0.054
**Received childbirth-related education during follow-up**	**No**	3.96 ± 1.60	4.10 ± 0.78	3.81 ± 0.94	3.74 ± 0.78	3.21 ± 0.85
	**Yes**	4.75 ± 1.60	4.43 ± 0.86	4.40 ± 1.01	4.02 ± 0.73	3.64 ± 0.88
	**Test / p / effect size**	U = 5866.500; p < 0.001; r = 0.208	U = 6175.000; p < 0.001; r = 0.187	U = 5350.500; p < 0.001; r = 0.256	U = 6585.500; p = 0.007; r = 0.151	U = 6134.000; p < 0.001; r = 0.187
**Attendance at antenatal education**	**No**	4.00 ± 1.59	4.12 ± 0.77	3.84 ± 0.92	3.77 ± 0.76	3.24 ± 0.87
	**Yes**	4.89 ± 1.71	4.47 ± 1.00	4.50 ± 1.18	4.00 ± 0.84	3.68 ± 0.84
	**Test / p / effect size**	U = 4038.500;	U = 4627.000;	U = 3783.500;	U = 4994.500;	U = 4249.000;

	p < 0.001; r = 0.193	p = 0.015; r = 0.138	p < 0.001; r = 0.223	p = 0.077; r = 0.100	p = 0.002; r = 0.175

Note. TIPI: Ten-Item Personality Inventory; SD: standard deviation; U: Mann–Whitney U test; KW: Kruskal–Wallis test. Mean ± SD values are presented descriptively. Because TIPI subscale scores were bounded two-item Likert-type scores and several subgroup sizes were unequal, exploratory subgroup comparisons were conducted using nonparametric tests. Mann–Whitney U tests were used for two-group comparisons, and Kruskal–Wallis tests were used for comparisons involving three or more groups. Effect sizes were reported as r for Mann–Whitney U tests and epsilon squared (ε^2) for Kruskal–Wallis tests. These analyses were exploratory and unadjusted. Personality-related subgroup findings should be interpreted cautiously, especially for Agreeableness, Emotional Stability, and Openness to Experience, which showed low internal consistency in the corrected reliability analysis. For “place of antenatal follow-up,” analyses were limited to women who had antenatal follow-up.
