# Supplementary figures and images for: Personality-Related Characteristics, Cultural Beliefs, and Labor Pain Perception After the 2023 Türkiye Earthquakes: A Prospective Study in Hatay
Source: Healthcare (Basel). 2026 Jun 23;14(13):1827. doi: 10.3390/healthcare14131827 (PMC13362373; doi:10.3390/healthcare14131827)

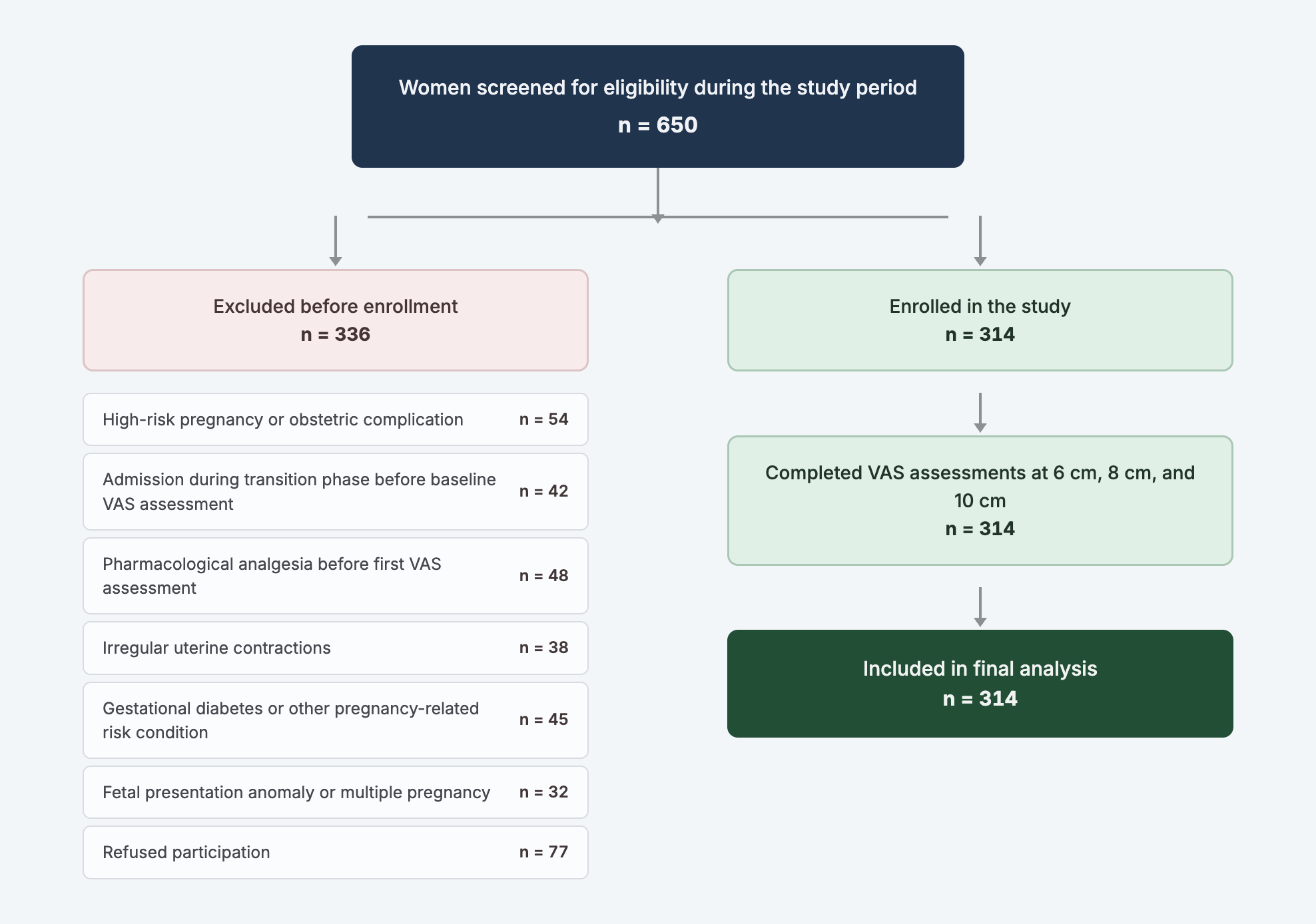

Supplement: Supplementary file 1 [file healthcare-14-01827-s001.zip › healthcare-4360334-supplementary/Supplementary Figure S1. Participant-flow diagram showing the number of women screened for eligibility, excluded before enrollme 1.png]
